# The Mediterranean diet is not associated with neuroimaging or cognition in middle‐aged adults: a cross‐sectional analysis of the PREVENT dementia programme

**DOI:** 10.1111/ene.16345

**Published:** 2024-05-25

**Authors:** Sarah Gregory, Ingrid Buller‐Peralta, Katie Bridgeman, Vanessa De La Cruz Góngora, Maria‐Eleni Dounavi, Audrey Low, Georgios Ntailianis, John O'Brien, Mario A. Parra, Craig W. Ritchie, Karen Ritchie, Oliver M. Shannon, Emma J. Stevenson, Graciela Muniz‐Terrera

**Affiliations:** ^1^ Edinburgh Dementia Prevention, Centre for Clinical Brain Sciences University of Edinburgh Edinburgh UK; ^2^ Scottish Brain Sciences Edinburgh UK; ^3^ Global Brain Health Institute, Institute of Neuroscience Trinity College Dublin Dublin Ireland; ^4^ Centre for Evaluation and Survey Research National Institute of Public Health Cuernavaca Mexico; ^5^ Department of Psychiatry, School of Clinical Medicine University of Cambridge Cambridge UK; ^6^ Department of Psychological Sciences and Health University of Strathclyde Glasgow UK; ^7^ Mackenzie Institute University of St Andrews St Andrews UK; ^8^ INM, Université de Montpellier, INSERM Montpellier France; ^9^ Human Nutrition and Exercise Research Centre, Population Health Sciences Institute, Faculty of Medical Sciences Newcastle University Newcastle upon Tyne UK; ^10^ Ohio University Heritage College of Osteopathic Medicine Ohio University Athens Ohio USA

**Keywords:** cognition, diet, midlife, neuroimaging

## Abstract

**Background and purpose:**

The Mediterranean diet (MedDiet) has been associated with reduced dementia incidence in several studies. It is important to understand if diet is associated with brain health in midlife, when Alzheimer's disease and related dementias are known to begin.

**Methods:**

This study used data from the PREVENT dementia programme. Three MedDiet scores were created (the Pyramid, Mediterranean Diet Adherence Screener [MEDAS] and MEDAS continuous) from a self‐reported food frequency questionnaire. Primary outcomes were hippocampal volume and cube‐transformed white matter hyperintensity volume. Secondary outcomes included cornu ammonis 1 and subiculum hippocampal subfield volumes, cortical thickness and measures of cognition. Sex‐stratified analyses were run to explore differential associations between diet and brain health by sex. An exploratory path analysis was conducted to study if any associations between diet and brain health were mediated by cardiovascular risk factors for dementia.

**Results:**

In all, 504 participants were included in this analysis, with a mean Pyramid score of 8.10 (SD 1.56). There were no significant associations between any MedDiet scoring method and any of the primary or secondary outcomes. There were no differences by sex in any analyses and no significant mediation between the Pyramid score and global cognition by cardiovascular risk factors.

**Conclusions:**

Overall, this study did not find evidence for an association between the MedDiet and either neuroimaging or cognition in a midlife population study. Future work should investigate associations between the MedDiet and Alzheimer's disease and related dementias biomarkers as well as functional neuroimaging in a midlife population.

## INTRODUCTION

Dietary patterns such as the Mediterranean diet (MedDiet) are effective at maintaining brain health [1]. The MedDiet is a plant‐based dietary pattern, characterized by high consumption of fruit, vegetables, olive oil, legumes, nuts and fish; moderate consumption of red wine; and low consumption of red meat, processed foods and sugar‐sweetened products [2]. In observational studies, high versus low MedDiet adherence has been associated with 10%–40% lower incidence of dementia, improved cognitive performance and less cortical atrophy [1, 3, 4, 5]. However, the evidence base is mixed and, although a MedDiet is recommended for dementia risk reduction by the World Health Organization (WHO), there is deemed to be insufficient evidence for diet to be included as one of the key modifiable risk factors for dementia in the Lancet Commission [6]. For further details of the differences in dietary recommendations in the WHO versus Lancet reports see Townsend et al. [7].

To date, most research investigating associations between diet, dementia and cognition has considered older adult populations [1, 3, 4, 5]. Alzheimer's disease and related dementias (ADRDs) are now known to have their origins in midlife [8], with many risk factors for ADRDs more predictive during midlife [6]. Given this, it is important to explore associations between diet and brain health in a midlife population.

Currently, there is limited evidence about associations between dietary patterns and neuroimaging correlates relevant to neurodegenerative conditions. Some studies reported associations between ‘healthy’ eating patterns and increased hippocampal volume [9], but the evidence specifically associating the MedDiet with hippocampal volume is weak [10]. Similarly, whilst some studies have identified associations between the MedDiet and white matter hyperintensity or lesion volume, the evidence remains inconclusive [9, 10]. Evidence also suggests poorer diet quality is associated with cortical thinning [4] and greater amyloid beta accumulation in the subiculum and cornu ammonis 1 (CA1) subfields.

Most studies have reported more significant associations between MedDiet and cardiovascular outcomes (e.g., insulin homeostasis, low‐density lipoprotein distribution and blood pressure) in male compared with female participants [11, 12, 13]. Typically, these studies have included older adult populations, reporting on interventional trials aimed at increasing adherence to the MedDiet. Interestingly, a recent analysis by our group using observational data from the PREVENT cohort found conversely that, in a midlife population, there were more significant associations between MedDiet adherence and cardiovascular health in female than male participants [14]. To date, there is little evidence of any sex‐specific associations between the MedDiet and brain health, with few studies reporting sex‐stratified results [15], and more evidence on this topic is needed.

This current analysis aimed to investigate associations between the MedDiet and measures of brain health (hippocampal volume, total and subfields, white matter hyperintensity volume [WMHV] and cognitive tests). The secondary analyses aimed to investigate differences by sex with the previously described outcome measures. It was hypothesized that (1) the MedDiet would be associated with more favourable brain health outcomes and that this would be mediated by cardiometabolic risk factors; (2) effects would be larger in female than male participants. Finally, exploratory analyses evaluated the mediating effect of cardiometabolic risk factors (systolic blood pressure [SBP], body mass index [BMI]) previously identified as associated with the MedDiet in the PREVENT cohort.

## METHODS

### PREVENT dementia programme

This cross‐sectional study used the baseline dataset (*n* = 700) from the PREVENT dementia programme [16, 17]. PREVENT recruited participants at five sites in the UK and Ireland (Cambridge, Dublin, Edinburgh, London and Oxford) who were aged 40–59 years, were free of dementia and just over half (52.6%) with a parental history of dementia (all‐cause). Participants were recruited from memory services which they attended as family members of a patient, from research registers, as well as from word of mouth. PREVENT was granted favourable ethical opinion by the London‐Camberwell St Giles National Health Service Research Ethics Committee (REC reference 12/LO/1023), the Trinity College Dublin School of Psychology Research Ethics Committee (SPREC022021‐010) and by the St James Hospital/Tallaght University Hospital Research Ethics Committee. Recruitment took place between 2014 and 2020 for the baseline cohort. All participants provided written informed consent prior to any protocol procedures. Baseline data are generally collected within a month of informed consent (typically, demographic, dietary and cognitive data collected on the date of consent and the magnetic resonance imaging [MRI] scans completed within a month).

### Calculation of diet scores

Three MedDiet scores (Pyramid, Mediterranean Diet Adherence Screener [MEDAS], MEDAS continuous) were calculated as previously described [14]. Briefly, MedDiet scores were calculated from the Scottish Collaborative Group Food Frequency Questionnaire, which gathered data on 175 different foods and drinks consumed by participants over the last 2–3 months. Full details of scoring methodologies are available in the supplementary material (Table S1). The MEDAS score awards points on a binary basis, with 1 point awarded for meeting the criteria for a food group, up to a total of 13 points within this dataset [18]. The MEDAS continuous score applies the same criteria on a continuous scale from 0 to 1, to award partial points to participants approaching criteria limits [19]. Finally, the Pyramid is another example of a continuous scoring methodology (up to 15 points within this dataset) which has been more widely used in the literature compared to the MEDAS continuous [20]. A Western diet score was created using a principal component analysis based on previously published methodology [21] and is fully described elsewhere [14]. The Western diet score included red meats, French fries, refined grains and snacks, with a higher score indicating great consumption of a Western diet (Table S2). Total energy intake (kcal/day) was derived from the dataset and included in the analysis. Participants with extreme energy intakes (<600 kcal, >6000 kcal) were excluded from the analysis (*n* = 1).

### Brain health variables

The primary outcome measure in this analysis was hippocampal volume derived from baseline structural MRI scans. All eligible participants (34 participants excluded due to previously unknown claustrophobia, metal implants or other medical history undisclosed at the time of study eligibility checking) underwent brain MRI using 3 T Siemens scanners (Skyra, Verio, Prisma and Prisma Fit). Hippocampal volume was derived from T1‐weighted structural scans using FreeSurfer Version 7.1.0 following correction for field inhomogeneities. In addition, the co‐primary analysis included total WMHV as quantified from fluid‐attenuated inversion recovery MRI using SPM12 (with all lesion masks visually inspected and manually corrected for segmentation errors, volumes normalized by total intracranial volume [TIV] to account for individual differences in head sizes and cube‐root transformed to account for significant right‐tailed skewness). Secondary analyses included deep and periventricular WMHV (both cube‐root transformed, quantified using the same methodology as described for total WMHV) and the following hippocampal subfields: CA1, subiculum, both derived from T1‐weighted structural scans using FreeSurfer Version 7.1.0. Further secondary analyses were tested for associations between diet and cortical thickness, also derived from T1‐weighted structural scans using FreeSurfer Version 7.1.0. More details on imaging acquisition and pre‐processing are available in previous publications [17, 22, 23, 24].

To supplement the neuroimaging measures, several cognitive test scores were included as secondary outcome measures. These included the Four Mountains Test (FMT) [25], the Visual Short‐Term Memory Binding Test (VSTMBT) [26] and the Addenbrookes Cognitive Examination III (ACE‐III) [27]. The FMT is a task of allocentric processing previously associated with risk for dementia both in the PREVENT cohort and with the MedDiet [28, 29]. Participants are asked to study a computer‐generated image of four hills for 10 s, following which the participants must select which of four new images shows the same topography as the initial target image, shown from a different viewpoint. Each correct answer is awarded 1 point, up to a total of 15 points, with higher scores indicating better performance. In the VSTMBT participants are presented with three visual stimuli, with a shape‐only condition and a colour–shape condition, and asked to recall after a brief delay whether the new screen shows the same or different shapes to the test condition. In the shape‐only condition, all shapes presented are black and participants are only recalling the shapes, whereas in the colour–shape condition participants are additionally recalling whether it is the same shape and the same colour, a process called memory binding. The binding cost was used in this current analysis, that is, the difference in score between the colour–shape condition and shape‐only condition, with higher binding cost indicating poorer performance on the colour–shape condition compared to shape only. The binding cost has been associated with amyloid‐beta burden in previous studies, and as such is a promising marker of early neurodegenerative disease [30]. Finally, the ACE‐III was selected as a measure of global cognition, providing a brief screen of memory, attention, fluency, language and visuospatial function.

### Calculating propensity scores

Propensity scores enable stronger conclusions on causality to be drawn in studies where random assignment is not possible, such as in PREVENT [31, 32]. Briefly, a propensity score is the probability that an individual would have been allocated a particular treatment group (in this case the MedDiet) as a function of observed baseline characteristics (as would be dealt with through a randomization process in a gold standard clinical trial). The propensity score is calculated using a linear regression model including variables that are theorized to relate to the treatment choice and/or outcome. The following variables were included in the generation of the propensity score: age (self‐reported), sex (self‐reported), years of education (self‐reported), parental history of dementia (self‐reported), *APOEε4* carrier status (genotype variant analysis carried out on QuantStudio12K Flex at the University of Edinburgh), socioeconomic status (based on occupational Office of National Statistics coding, categorized as high, moderate and low socioeconomic status) and physical activity (self‐reported based on the frequency of engaging in low, moderate and vigorous activity). These variables were selected based on a review of the literature of factors associated with MedDiet adherence [33, 34, 35, 36, 37] and to replicate the approach taken in the early analysis investigating associations between the MedDiet and cardiovascular risk factors [14].

### Statistical analysis

All statistical analyses were completed using R (version 4.1.0). Descriptive statistics were calculated for all participants. For the main analysis, participants with missing data in the exposure, outcome and covariate variables of interest from the analysis were excluded. For each outcome, the same analytical steps were followed. First, the cohort as a whole was tested and univariate and fully adjusted linear regression models were fitted to test for associations between Pyramid scores and brain health outcomes. Analyses of hippocampal volume, CA1 and subiculum hippocampal subfields were corrected for estimated TIV through inclusion as a covariate in the linear regression models. Total, deep and periventricular WMHVs were corrected for TIV prior to cube‐root transformation. The partially adjusted model included energy intake (kcal/day) and the propensity score as covariates. In the fully adjusted model, the Western diet score was also added as a covariate. The analysis was then repeated with the MEDAS and MEDAS continuous scores instead of the Pyramid score as the measure of MedDiet adherence. The Pyramid score was selected as the primary exposure variable based on the earlier analysis in this same cohort. Results were corrected using the Benjamini–Hochberg false discovery rate (FDR) procedure to adjust for multiple comparisons. Based on the results of the analysis of associations between MedDiet and cardiovascular health in the PREVENT cohort [14], our pre‐planned stratified analysis split the dataset into male and female participants and re‐ran the same model using the Pyramid score as the exposure variable of interest. Finally, an exploratory path analysis was run to understand whether any significant associations were in part mediated by either BMI or SBP.

## RESULTS

### Descriptive statistics

In all, 516 participants were included in the primary analysis of WMHV data and 504 participants in the primary analysis of hippocampal volume data. Participants were excluded if they had missing hippocampal volume or WMHV data, or if the scans did not pass quality control (QC) (WHMV missing MRI *n* = 34, missing WMHV *n* = 37, did not pass QC *n* = 13; missing dietary data *n* = 99 or implausible calorie intake *n* = 1; hippocampal volume missing MRI *n* = 34, missing hippocampal volume *n* = 23, did not pass QC *n* = 33; missing dietary data *n* = 105 or an implausible calorie intake *n* = 1). Differences between key demographic data and Pyramid scores for participants included compared to excluded are presented in Table 1. Secondary analyses included different numbers of participants depending on the data availability, and the sample size available for each variable is detailed in Table 2. Descriptive statistics are calculated from the WMHV analytical dataset. Participants included in the sample had a mean age of 51.23 (±5.42) years, were highly educated (mean of 16.70 ± 3.31 years of education), with a majority being female (*n* = 310, 60.0%), with a parental history of dementia (*n* = 272, 52.7%) and 38.4% (*n* = 198) of the sample were *APOEε4* carriers. Participants had a moderate adherence to the MedDiet as quantified by the Pyramid (8.09 [±1.55]), MEDAS (5.43 [±1.74]) and MEDAS continuous (7.28 [±1.59]) scores. Participants were all cognitively normal according to their ACE‐III scores and whilst female participants scored statistically significantly higher on the ACE‐III compared to male participants this approximately 1‐point difference is unlikely to be clinically meaningful. For full demographic and descriptive details see Table 2.

**TABLE 1:** Descriptive statistics for participants included in the WMHV analysis (*n* = 516) versus excluded (*n* = 184) and in the hippocampal volume analysis (*n* = 504) versus excluded (*n* = 196) and an indication of any significant differences between included and excluded participants.

Variable	WHMV analysis			Hippocampal volume analysis		
	Included: mean (SD)/*N* (%)	Excluded: mean (SD)/*N* (%)	Significance tests	Included: mean (SD)/*N* (%)	Excluded: mean (SD)/*N* (%)	Significance tests
Age (years)	51.13 (5.42)	51.01 (5.62)	*t* = −0.48, *p* = 0.63	51.18 (5.38)	51.15 (5.73)	*t* = −0.07, *p* = 0.94
Education (years)	16.75 (3.60)	16.70 (3.31)	*t* = 0.17, *p* = 0.86	16.73 (3.27)	16.68 (3.68)	*t* = −0.17, *p* = 0.86
Sex (female)	310 (60.1)	123 (66.8)	*X* ^2^ = 2.79, *p* = 0.09	303 (60.1)	130 (66.3)	*X* ^2^ = 2.30, *p* = 0.13
*APOEε4 c*arriers Non‐carrier N/A	198 (38.4) 318 (61.6) 0 (0)	65 (35.1) 110 (59.5) 10 (5.4)	*X* ^2^ = 28.38, *p* < 0.001	192 (38.1) 312 (61.9) 0 (0)	71 (36.2) 116 (59.2) 9 (4.6)	*X* ^2^ = 23.45, *p* < 0.001
Parental history of dementia (yes)	88 (47.6)	272 (52.7)	*X* ^2^ = 1.44, *p* = 0.23	265 (52.6)	95 (48.5)	*X* ^2^ = 0.95, *p* = 0.33
Pyramid	8.16 (1.40)	8.09 (1.55)	*t* = 0.45, *p* = 0.65	8.10 (1.56)	8.12 (1.37)	*t* = 0.12, *p* = 0.91

Abbreviation: WHMV, white matter hyperintensity volume.

**TABLE 2:** Descriptive statistics for participants included in the analysis (*n* = 516), with sex stratification and an indication of any significant differences between male and female participants.

Variable	Mean (SD)/*N* (%)	Female *n* = 310	Male *n* = 206	Significance tests by sex
Age (years)	51.23 (5.42)	50.85 (5.31)	51.81 (5.55)	*t* = −1.98, *p* = 0.05
Education (years)	16.70 (3.31)	16.94 (3.55)	16.34 (2.90)	*t* = 2.03, *p* = 0.04
Sex (female)	310 (60.0)			
*APOEε4 c*arriers	198 (38.4)	121 (39.0)	77 (37.4)	*X* ^2^ = 0.14, *p* = 0.71
Parental history of dementia	272 (52.7)	168 (54.2)	104 (50.5)	*X* ^2^ = 0.68, *p* = 0.41
Ethnicity (Caucasian)	496 (96.1)	297 (95.8)	199 (96.6)	*X* ^2^ = 6.05, *p* = 0.20
SES group Low Middle High Unemployed	38 (7.4) 82 (15.9) 333 (64.5) 63 (12.2)	21 (6.8) 58 (18.7) 195 (62.9) 36 (11.6)	17 (8.3) 24 (11.7) 27 (13.1) 138 (67.0)	*X* ^2^ = 4.79, *p* = 0.19
Physical activity score	10.88 (2.85)	10.59 (2.84)	11.38 (2.69)	*t* = −3.37, *p* = 0.0008
*Diet scores*				
Pyramid	8.09 (1.55)	8.34 (1.60)	7.73 (1.39)	*t* = 4.42, *p* < 0.001
MEDAS	5.43 (1.74)	5.61 (1.78)	5.17 (1.63)	*t* = 2.87, *p* = 0.004
MEDAS continuous	7.28 (1.59)	7.44 (1.60)	7.03 (1.54)	*t* = 2.94, *p* = 0.003
Western diet score	4.90 (2.63)	4.57 (2.63)	5.39 (2.56)	*t* = −3.51, *p* = 0.0005
Calories (kcal)	2034.61 (759.68)	1937.41 (738.36)	2180.88 (769.60)	*t* = −3.61, *p* = 0.0003
*Brain health variables*				
Mean hippocampal volume^a^	4102.27 (391.20)	3973.01 (333.97)	4297.12 (391.16)	*t* = −9.96, *p* < 0.001
Total WMHV (% of TIV)	0.13 (0.18)	0.11 (0.16)	0.17 (0.21)	*t* = −3.87, *p* = 0.0001
Deep WHMV (% of TIV)	0.05 (0.07)	0.04 (0.06)	0.07 (0.09)	*t* = −4.00, *p* < 0.001
Periventricular WMHV (% of TIV)	0.08 (0.12)	0.07 I0.11)	0.11 (0.13)	*t* = −3.37, *p* = 0.0008
CA1^b^	1372.30 (147.53)	1325.69 (130.16)	1443.39 (144.38)	*t* = −9.00, *p* < 0.001
Subiculum^b^	1624.62 (161.86)	1580.41 (147.01)	1692.05 (160.58)	*t* = −7.61, *p* < 0.001
Cortical thickness^c^	2.43 (0.06)	2.43 (0.06)	2.44 (0.07)	*t* = −1.01, *p* = 0.32
Four Mountains Test total score^d^	10.47 (2.39)	10.33 (2.38)	10.62 (2.40)	*t* = −1.10, *p* = 0.27
ACE‐III total score^d^	96.02 (3.58)	96.67 (3.06)	95.27 (3.98)	*t* = 3.61, *p* < 0.001
Binding cost^e^	18.92 (12.16)	19.12 (12.86)	18.61 (11.03)	*t* = 0.46, *p* = 0.65
*Cardiovascular mediating variables*				
BMI^f^	27.54 (5.51)	27.22 (6.57)	27.92 (3.92)	*t* = −1.14, *p* = 0.25
SBP^f^	127.23 (15.59)	122.34 (14.86)	132.91 (14.48)	*t* = −6.53, *p* < 0.001

Abbreviations: ACE‐III, Addenbrookes Cognitive Examination III; BMI, body mass index; CA1, cornu ammonis 1; MEDAS, Mediterranean Diet Adherence Screener; SBP, systolic blood pressure; SES, socioeconomic status; TIV, total intracranial volume; WHMV, white matter hyperintensity volume.

^a^ *n* = 504, female *n* = 303, male *n* = 201.

^b^ *n* = 452, female *n* = 273, male *n* = 179.

^c^ *n* = 502, female *n* = 303, male *n* = 199.

^d^ *n* = 331, female *n* = 178, male *n* = 153.

^e^ *n* = 505, female *n* = 303, male *n* = 201.

^f^ *n* = 331, female *n* = 178, male *n* = 153.

### Associations between MedDiet, white matter hyperintensity volume and hippocampal volume

There were no significant associations between any of the MedDiet scoring approaches and either cube‐transformed WMHV or TIV normalized hippocampal volume in any of the models (Pyramid fully adjusted cube‐root transformed WMHV *β* = −0.006, SE 0.004, *p* = 0.13; Pyramid fully adjusted hippocampal volume *β* = −15.53, SE 9.64, *p* = 0.11). Full information is presented in Table 3.

**TABLE 3:** Associations between dietary scores, primary and secondary outcomes.

Dietary score	Model 1	Model 2	Model 3
Co‐primary outcomes			
Cube‐root transformed total white matter hyperintensity volume (*n* = 516)			
Pyramid	*β* = −0.006, SE 0.004, *p* = 0.13	*β* = −0.003, SE 0.004, *p* = 0.49	*β* = −0.005, SE 0.005, *p* = 0.25
MEDAS	*β* = −0.002, SE 0.003, *p =* 0.58	*β* = −0.0006, SE 0.004, *p* = 0.88	*β* = −0.002, *p* 0.004, *p* = 0.57
MEDAS continuous	*β* = −0.004, SE 0.004, *p* = 0.37	*β* = −0.0007, SE 0.004, *p* = 0.87	*β* = −0.005, SE 0.005, *p* = 0.35
Mean hippocampal volume (*n* = 504)			
Pyramid	*β* = −14.19, SE 8.75, *p* = 0.11	*β* = −13.50, SE 9.20, *p* = 0.14	*β* = −15.53, SE 9.64, *p* = 0.11
MEDAS	*β* = −3.29, SE 7.96, *p* = 0.68	*β* = −1.87, SE 8.35, *p* = 0.82	*β* = −3.65, SE 9.53, *p* = 0.70
MEDAS continuous	*β* = −2.09, SE 8.66, *p* = 0.81	*β* = −0.28, SE 9.12, *p* = 0.98	*β* = −1.70, SE 10.41, *p* = 0.87
Secondary outcomes			
CA1 volume (*n* = 452)			
Pyramid	*β* = −6.97, SE 3.58, *p* = 0.05	*β* = −7.72, SE 3.70, *p* = 0.04	*β* = −9.05, SE 3.89, *p* = 0.02
MEDAS	*β* = −5.79, SE 3.17, *p* = 0.07	*β* = −6.58, SE 3.29, *p* = 0.05	*β* = −9.85, SE 3.82, *p* = 0.01
MEDAS continuous	*β* = −4.39, SE 3.50, *p* = 0.21	*β* = −5.16, SE 3.65, *p* = 0.16	*β* = −8.03, SE 4.24, *p* = 0.06
Subiculum volume (*n* = 452)			
Pyramid	*β* = 2.06, SE 4.08, *p* = 0.61	*β* = 2.37, SE 4.22, *p* = 0.58	*β* = 1.63, SE 4.45, *p* = 0.71
MEDAS	*β* = 0.23, SE 3.61, *p* = 0.95	*β* = 0.36, SE 3.75, *p* = 0.92	*β* = −1.30, SE 4.37, *p* = 0.77
MEDAS continuous	*β* = 2.23, SE 3.98, *p* = 0.58	*β* = 2.56, SE 4.15, *p* = 0.54	*β* = 1.49, SE 4.84, *p* = 0.76
Cortical thickness (*n* = 502)			
Pyramid	*β* = 0.0006, SE 0.002, *p* = 0.74	*β* = 0.001, SE 0.002, *p* = 0.57	*β* = 0.001, SE 0.002, *p* = 0.52
MEDAS	*β* = −0.0003, SE 0.002, *p* = 0.87	*β* = −0.00003, SE 0.003, *p* = 0.99	*β* = 0.0002, SE 0.002, *p* = 0.91
MEDAS continuous	*β* = 0.0002, SE 0.002, *p* = 0.92	*β* = 0.0006, SE 0.002, *p* = 0.76	*β* = 0.001, SE 0.002, *p* = 0.65
Cube‐root transformed deep white matter hyperintensity volume (*n* = 504)			
Pyramid	*β* = −0.005, SE 0.003, *p* = 0.10	*β* = −0.003, SE 0.003, *p* = 0.41	*β* = −0.004, SE 0.004, *p* = 0.24
MEDAS	*β* = −0.002, SE 0.003, *p* = 0.48	*β* = −0.0002, SE 0.003, *p* = 0.95	*β* = −0.002, SE 0.003, *p* = 0.65
MEDAS continuous	*β* = −0.004, SE 0.003, *p* = 0.25	*β* = −0.001, SE 0.003, *p* = 0.71	*β* = −0.004, SE 0.004, *p* = 0.33
Cube‐root transformed periventricular white matter hyperintensity volume (*n* = 504)			
Pyramid	*β* = −0.005, SE 0.004, *p* = 0.18	*β* = −0.003, SE 0.004, *p* = 0.50	*β* = −0.005, SE 0.004, *p* = 0.23
MEDAS	*β* = −0.001, SE 0.004, *p* = 0.70	*β* = 0.0006, SE 0.004, *p* = 0.87	*β* = −0.003, SE 0.004, *p* = 0.52
MEDAS continuous	*β* = −0.003, SE 0.004. *p* = 0.45	*β* = −0.0006, SE 0.004, *p* = 0.88	*β* = −0.005, SE 0.005, *p* = 0.30
Four Mountains Test (*n* = 331)			
Pyramid	*β* = 0.09, SE 0.09, *p* = 0.30	*β* = 0.04, SE 0.09, *p* = 0.67	*β* = 0.02, SE 0.10, *p* = 0.87
MEDAS	*β* = 0.08, SE 0.08, *p* = 0.32	*β* = 0.05, SE 0.08, *p* = 0.55	*β* = 0.02, SE 0.10, *p* = 0.85
MEDAS continuous	*β* = 0.09, SE 0.09, *p* = 0.30	*β* = 0.05, SE 0.09, *p* = 0.57	*β* = 0.01, SE 0.11, *p* = 0.89
ACE‐III (*n* = 331)			
Pyramid	*β* = 0.44, SE 0.13, *p* = 0.0008	*β* = 0.37, SE 0.14, *p* = 0.007	*β* = 0.35, SE 0.15, *p* = 0.02
MEDAS	*β* = 0.19, SE 0.12, *p* = 0.11	*β* = 0.13, SE 0.12, *p* = 0.28	*β* = 0.06, SE 0.14, *p* = 0.68
MEDAS continuous	*β* = 0.31, SE 0.13, *p* = 0.02	*β* = 0.24, SE 0.13, *p* = 0.07	*β* = 0.20, SE 0.16, *p* = 0.21
Binding cost (*n* = 505)			
Pyramid	*β* = 0.63, SE 0.35, *p* = 0.07	*β* = 0.55, SE 0.36, *p* = 0.13	*β* = 0.52, SE 0.39, *p* = 0.18
MEDAS	*β* = 0.51, SE 0.31, *p* = 0.11	*β* = 0.43, SE 0.33, *p* = 0.19	*β* = 0.40, SE 0.38, *p* = 0.29
MEDAS continuous	*β* = 0.61, SE 0.34, *p* = 0.07	*β* = 0.54, SE 0.36, *p* = 0.14	*β* = 0.53, SE 0.41, *p* = 0.20

Note: Model 1, adjusted for estimated intracranial volume (except for Four Mountains Test, ACE‐III and binding cost); model 2, additionally adjusted for propensity score and total kilocalories; model 3, additionally adjusted for Western diet score.

Abbreviations: ACE‐III, Addenbrookes Cognitive Examination III; CA1, cornu ammonis 1; MEDAS, Mediterranean Diet Adherence Screener.

### Associations between MedDiet, hippocampal subfields, cortical thickness, deep and periventricular WMHV

**FIGURE 1:**
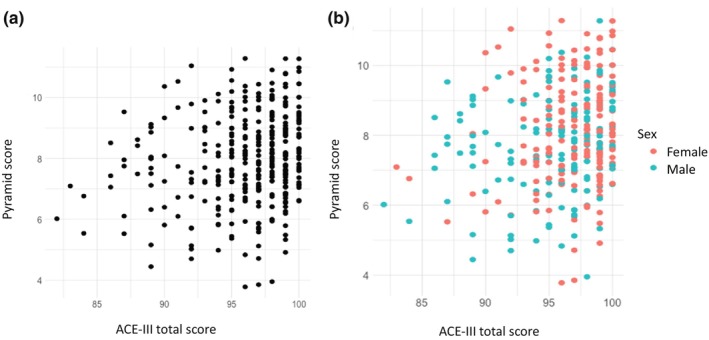
Scatterplot of Pyramid score with ACE‐III total score. (a) The data in the cohort as a whole. (b) Sex‐stratified information. ACE‐III: Addenbrookes Cognitive Examination III.

There was a significant association between higher adherence to the MedDiet in fully adjusted models where the Pyramid and MEDAS scores were used and lower CA1 subfield volume. However, neither remained significant after FDR adjustment (Pyramid fully adjusted *β* = −9.05, SE 3.89, *p* = 0.02; MEDAS fully adjusted *β* = −9.85, SE 3.82, *p* = 0.01) (see Table 3 and Figure 1). There were no significant associations between any MedDiet scoring methodology and subiculum volume, cortical thickness, cube‐root transformed deep or periventricular WMHV (Pyramid fully adjusted subiculum *β* = 1.63, SE 4.45, *p* = 0.71; Pyramid fully adjusted cortical thickness *β* = 0.001, SE 0.002, *p* = 0.52; Pyramid fully adjusted deep WMHV *β* = −0.005, SE 0.003, *p* = 0.10; Pyramid fully adjusted periventricular WMHV *β* = −0.005, SE 0.004, *p* = 0.18).

### Associations between MedDiet and cognition

Adherence to the MedDiet as measured by the Pyramid score was significantly associated with higher ACE‐III scores, representing global cognition, although only the unadjusted model remained statistically significant after FDR adjustment (unadjusted *β* = 0.44, SE 0.13, *p* = 0.0008; partially adjusted *β* = 0.37, SE 0.14, *p* = 0.007; fully adjusted *β* = 0.35, SE 0.15, *p* = 0.02). There were no statistically significant associations between any MedDiet scoring method and either FMT total score or the memory binding cost (Pyramid fully adjusted FMT *β* = 0.02, SE 0.10, *p* = 0.87; Pyramid fully adjusted binding cost *β* = 0.52, SE 0.39, *p* = 0.18).

### Associations between MedDiet and brain health: sex‐stratified analyses

There were no significant associations between MedDiet adherence as quantified by the Pyramid score and any of the brain health outcomes in fully adjusted models when female and male participants were considered separately (Table 4). There was a significant association between higher Pyramid scores and higher FMT scores in female participants in the unadjusted model. However, this was attenuated by the addition of the propensity score to the partially and fully adjusted models. There was also a significant association between higher Pyramid scores and higher cortical thickness in the unadjusted model, and between higher Pyramid scores and higher ACE‐III scores in male participants in the unadjusted and partially adjusted models; however, neither association was significant in the fully adjusted models (see unadjusted association between Pyramid scores and ACE‐III total scores by sex in Figure 1).

**TABLE 4:** Associations between dietary scores, primary and secondary outcomes by sex.

Dietary score	Female			Male		
	Model 1	Model 2	Model 3	Model 1	Model 2	Model 3
Co‐primary outcomes						
Cube‐root transformed total white matter hyperintensity volume^a^						
Pyramid	*β* = −0.001, SE 0.005, *p* = 0.82	*β* = −0.0009, SE 0.005, *p* = 0.86	*β* = −0.0007, SE 0.005, *p* = 0.89	*β* = −0.003, SE 0.008, *p* = 0.66	*β* = −0.002, SE 0.008, *p* = 0.79	*β* = −0.005, SE 0.008, *p* = 0.55
Mean hippocampal volume^b^						
Pyramid	*β* = −6.26, SE 10.70, *p* = 0.56	*β* = −4.00, SE 11.03, *p* = 0.72	*β* = −3.42, SE 11.91, *p* = 0.77	*β* = −21.15, SE 16.24, *p* = 0.19	*β* = −27.79, SE 16.62, *p* = 0.10	*β* = −31.78, SE 16.96, *p* = 0.06
Secondary outcomes						
CA1 volume^c^						
Pyramid	*β* = −6.30, SE 4.48, *p* = 0.16	*β* = −7.24, SE 4.53, *p* = 0.11	*β* = −8.22, SE 4.95, *p* = 0.10	*β* = −6.86, SE 6.56, *p* = 0.30	*β* = −8.62, SE 6.69, *p* = 0.20	*β* = −9.69, SE 6.84, *p* = 0.16
Subiculum volume^c^						
Pyramid	*β* = −0.05, SE 5.16, *p* = 0.99	*β* = −0.27, SE 5.23, *p* = 0.96	*β* = −0.56, SE 5.72, *p* = 0.92	*β* = 6.73, SE 7.37, *p* = 0.36	*β* = 6.21, SE 7.54, *p* = 0.41	*β* = 5.60, SE 7.71, *p* = 0.47
Cortical thickness^d^						
Pyramid	*β* = −0.002, SE 0.002, *p* = 0.30	*β* = −0.001, SE 0.002, *p* = 0.49	*β* = −0.002, SE 0.002, *p* = 0.49	*β* = 0.007, SE 0.003, *p* = 0.03	*β* = 0.006, SE 0.003, *p* = 0.08	*β* = 0.006, SE 0.004, *p* = 0.08
Cube‐root transformed deep white matter hyperintensity volume^b^						
Pyramid	*β* = −0.003, SE 0.004, *p* = 0.48	*β* = −0.002, SE 0.004, *p* = 0.61	*β* = −0.002, SE 0.004, *p* = 0.72	*β* = −0.0007, SE 0.006, *p* = 0.91	*β* = −0.0004, SE 0.006, *p* = 0.95	*β* = −0.002, SE 0.006, *p* = 0.72
Cube‐root transformed periventricular white matter hyperintensity volume^b^						
Pyramid	*β* = 0.0002, SE 0.005, *p* = 0.97	*β* = −0.0002, SE 0.005, *p* = 0.97	*β* = −0.0003, SE 0.005, *p* = 0.96	*β* = −0.005, SE 0.007, *p* = 0.47	*β* = −0.004, SE 0.008, *p* = 0.61	*β* = −0.007, SE 0.008, *p* = 0.38
Four Mountains Test^e^						
Pyramid	*β* = 0.27, SE 0.12, *p* = 0.02	*β* = 0.18, SE 0.12, *p* = 0.14	*β* = 0.17, SE 0.13, *p* = 0.20	*β* = −0.11, SE 0.14, *p* = 0.44	*β* = −0.15, SE 0.15, *p* = 0.31	*β* = −0.17, SE 0.15, *p* = 0.28
ACE‐III^e^						
Pyramid	*β* = 0.26, SE 0.16, *p* = 0.09	*β* = 0.26, SE 0.16, *p* = 0.11	*β* = 0.29, SE 0.17, *p* = 0.10	*β* = 0.55, SE 0.23, *p* = 0.02	*β* = 0.49, SE 0.24, *p* = 0.04	*β* = 0.39, SE 0.25, *p* = 0.12
Binding cost^f^						
Pyramid	*β* = 0.93, SE 0.46, *p* = 0.05	*β* = 0.88, SE 0.48, *p* = 0.07	*β* = 0.67, SE 0.51, *p* = 0.19	*β* = 0.02, SE 0.56, *p* = 0.97	*β* = −0.02, SE 0.59, *p* = 0.97	*β* = 0.09, SE 0.60, *p* = 0.87

Note: Model 1, adjusted for estimated intracranial volume (except for Four Mountains Test, ACE‐III and binding cost); model 2, additionally adjusted for propensity score (without sex) and total kilocalories; model 3, additionally adjusted for Western diet score.

Abbreviations: ACE‐III, Addenbrookes cognitive examination III; CA1, cornu ammonis 1.

^a^ Female *n* = 310, male *n* = 206.

^b^ Female *n* = 303, male *n* = 201.

^c^ Female *n* = 273, male *n* = 179.

^d^ Female *n* = 303, male *n* = 199.

^e^ Female *n* = 178, male *n* = 153.

^f^ Female *n* = 303, male *n* = 202.

#### Exploratory mediation analyses

Exploratory pathway analyses were investigated if the uncorrected associations between the Pyramid score and ACE‐III score could be explained by either BMI or SBP, given previous significant associations between these two variables and MedDiet adherence in the PREVENT cohort. Although the Pyramid score was significantly associated with total ACE‐III score and with both BMI and SBP, neither was identified as a significant mediator between the Pyramid score and total ACE‐III score (see Figure S1).

## DISCUSSION

There were no significant associations between adherence to the MedDiet and either primary outcome measure of hippocampal volume or WMHV in middle‐aged adults in PREVENT. There was a significant association between higher Pyramid scores and higher ACE‐III scores in all models; however, only the unadjusted model remained significant following correction for multiple comparisons. This association was not mediated by either BMI or SBP. There were no notable differences in MedDiet and brain health associations by sex.

Our aim was to explore whether there are associations between MedDiet and neuroimaging and cognition in midlife. Our results suggest that MedDiet is not associated with these measures in midlife. In the context of known associations with better cardiovascular health in a midlife population [14], it may be that any benefits conferred for reduced dementia risk in later life [1, 3, 4, 5, 29, 38] are through reduced cardiovascular burden in midlife. The MedDiet adherence scores are in line with other UK study populations [19, 39] and therefore our lack of significant results for the brain health outcome measures are unlikely to be related to significant under‐consumption of the MedDiet. There were no significant associations in this current study in the exploratory path analysis, suggesting any cognitive benefits that may arise from better cardiovascular health are not evident in this midlife population. Given the participants in the PREVENT cohort are an estimated 24 years from predicted dementia onset at baseline [40], it is also possible that any changes in neuroimaging or cognition are too subtle to identify any associations with dietary patterns at this stage. Considering the hypothetical model of Alzheimer's disease (AD) posited by Jack et al., structural neuroimaging and cognitive changes appear as later‐stage events in the AD process, with the accumulation of amyloid and tau pre‐dating these changes [41, 42]. Previous studies have shown evidence of associations between a MedDiet and reduced AD biomarker burden in older adults [43, 44] and postmortem [45]. These potential associations between the diet and the earliest stages of pathophysiology warrant further investigation where ADRD biomarkers are available in midlife cohorts [46].

Additionally, investigating associations between the MedDiet and functional neuroimaging in midlife populations is an avenue for future research efforts. Recent studies have reported significant associations between the MedDiet and increased cerebral perfusion [46], higher fractional anisotropy, higher structural connectivity in the amygdala, lingual, olfactory, middle occipital gyrus and calcarine areas and lower diffusivity in white matter [47]. Low adherence to the MedDiet has also been reported to moderate the relationship between resting state functional connectivity and performance on a test of fluid reasoning [48]. This emerging evidence base suggests that further investigation of associations between MedDiet and functional/physiological neuroimaging measures may help to identify any mechanistic explanations in midlife that may explain later associations with lower dementia incidence.

Whilst this current study explored any differential associations between diet and brain health by sex, it was not possible to consider the role of gender. Although most previous research has considered the role of sex [11, 12, 13, 14, 15], gender may actually be the more relevant construct to consider in relation to dietary adherence [15]. Many foods have been traditionally associated with either masculinity (e.g., meat) or femininity (e.g., vegetables, dairy) highlighting the potential for gender to influence dietary choices [15, 49], and further research is needed to unpick associations of sex and gender with diet and health outcomes separately.

There are some limitations to this study that should be noted. The Food Frequency Questionnaire used to collect the dietary data was administered as a self‐report questionnaire with the potential for biases in the reporting through underestimation of food intake compared to more detailed methodologies such as a food diary [50]. In addition, the period of diet collection referred to 2–3 months, which might not capture the habitual diet (the usual intake of foods during the year) being influenced by seasonality. There were also several participants who were excluded from the secondary analyses of the ACE‐III and FMT, resulting in a relatively small sample size, particularly when the data were stratified by sex. Both tasks were added into the protocol through a protocol amendment and as such only participants who enrolled in the study after 2016 have these data points available. Finally, it should be noted that the participants included in this sample were highly educated and included very little ethnic diversity (96.2% Caucasian) which does not accurately reflect the UK and Irish populations. Further research in studies that are more representative of the UK and Irish populations on key demographics is required.

This study did not identify any cross‐sectional associations between MedDiet and brain health in a midlife population living in the UK and Ireland. Future work could consider investigating ADRD biomarkers and functional neuroimaging to understand whether there are associations between MedDiet and the earliest neurodegenerative pathophysiology.

## AUTHOR CONTRIBUTIONS

**Sarah Gregory:** Conceptualization; investigation; writing – original draft; methodology; writing – review and editing; formal analysis; project administration; data curation. **Ingrid Buller‐Peralta:** Writing – review and editing. **Katie Bridgeman:** Methodology; writing – review and editing. **Vanessa De La Cruz Góngora:** Writing – review and editing. **M Dounavi:** Writing – review and editing. **Audrey Low:** Writing – review and editing. **Georgios Ntailianis:** Writing – review and editing. **John O'Brien:** Writing – review and editing. **Mario A. Parra:** Writing – review and editing. **Craig W. Ritchie:** Funding acquisition; writing – review and editing; supervision. **Karen Ritchie:** Writing – review and editing. **Oliver M. Shannon:** Writing – review and editing; methodology; conceptualization. **Emma J. Stevenson:** Writing – review and editing. **Graciela Muniz‐Terrera:** Conceptualization; writing – review and editing; methodology; supervision.

## FUNDING INFORMATION

The PREVENT dementia programme is funded by the Alzheimer's Society (grant numbers 178, 264 and 329), Alzheimer's Association (grant number TriBEKa‐17‐519,007) and philanthropic donations. The analytical work was funded by the MRC UK Nutrition Research Partnership (NRP) Collaboration Award (MR/T001852/1). Professor Muniz‐Terrera acknowledges the support of the Osteopathic Heritage Foundation through funding for the Osteopathic Heritage Foundation Ralph S. Licklider, D.O., Research Endowment in the Heritage College of Osteopathic Medicine. The funders had no involvement in the protocol design, data collection, analysis or manuscript preparation. For the purpose of open access, the authors have applied a Creative Commons attribution (CC BY) licence to any author‐accepted manuscript version arising.

## CONFLICT OF INTEREST STATEMENT

The authors declare that the research was conducted in the absence of any commercial or financial relationships that could be construed as a potential conflict of interest. SG is funded by the MRC UK Nutrition Research Partnership (NRP) Collaboration Award (MR/T001852/1). SG additionally received salary from Scottish Brain Sciences. GN and KW are funded by the PREVENT study funders (Alzheimer's Society, grant numbers 178, 264 and 329; Alzheimer's Association, grant number TriBEKa‐17‐519,007; and philanthropic donations). John O'Brien has no conflicts related to this work. Outside of this work he has acted as a consultant for TauRx, Novo Nordisk, Biogen, Roche, Lilly and GE Healthcare and received grant or academic support from Avid/Lilly, Merck and Alliance Medical. CWR is the CEO and founder of Scottish Brain Sciences and has previously received consulting fees from Biogen, Eisai, MSD, Actinogen, Roche and Eli Lilly, as well as receiving speaker fees from Roche and Eisai. CWR sits on an NIHR data safety monitoring board and is on an advisory board for Roche Diagnostics. CWR is an unpaid chair of the Brain Health Clinic Consortium (sponsored by Biogen). No other conflicts of interest are declared by other authors.

## CONSENT STATEMENT

All participants provided informed consent prior to undertaking any protocol‐related activities.

## Supporting information


**APPENDIX S1:** Supporting Information.

**APPENDIX S2:** Supporting Information.

## Data Availability

The data that support the findings of this study are openly available in PREVENT Main Baseline 700 V1 at https://www.alzheimersdata.org/ad‐workbench, reference number https://doi.org/10.34688/PREVENTMAIN_BASELINE_700V1.
