# Supplementary figures and images for: The Mediterranean diet is not associated with neuroimaging or cognition in middle‐aged adults: a cross‐sectional analysis of the PREVENT dementia programme
Source: Eur J Neurol. 2024 May 25;31(8):e16345. doi: 10.1111/ene.16345 (PMC11236004; doi:10.1111/ene.16345)

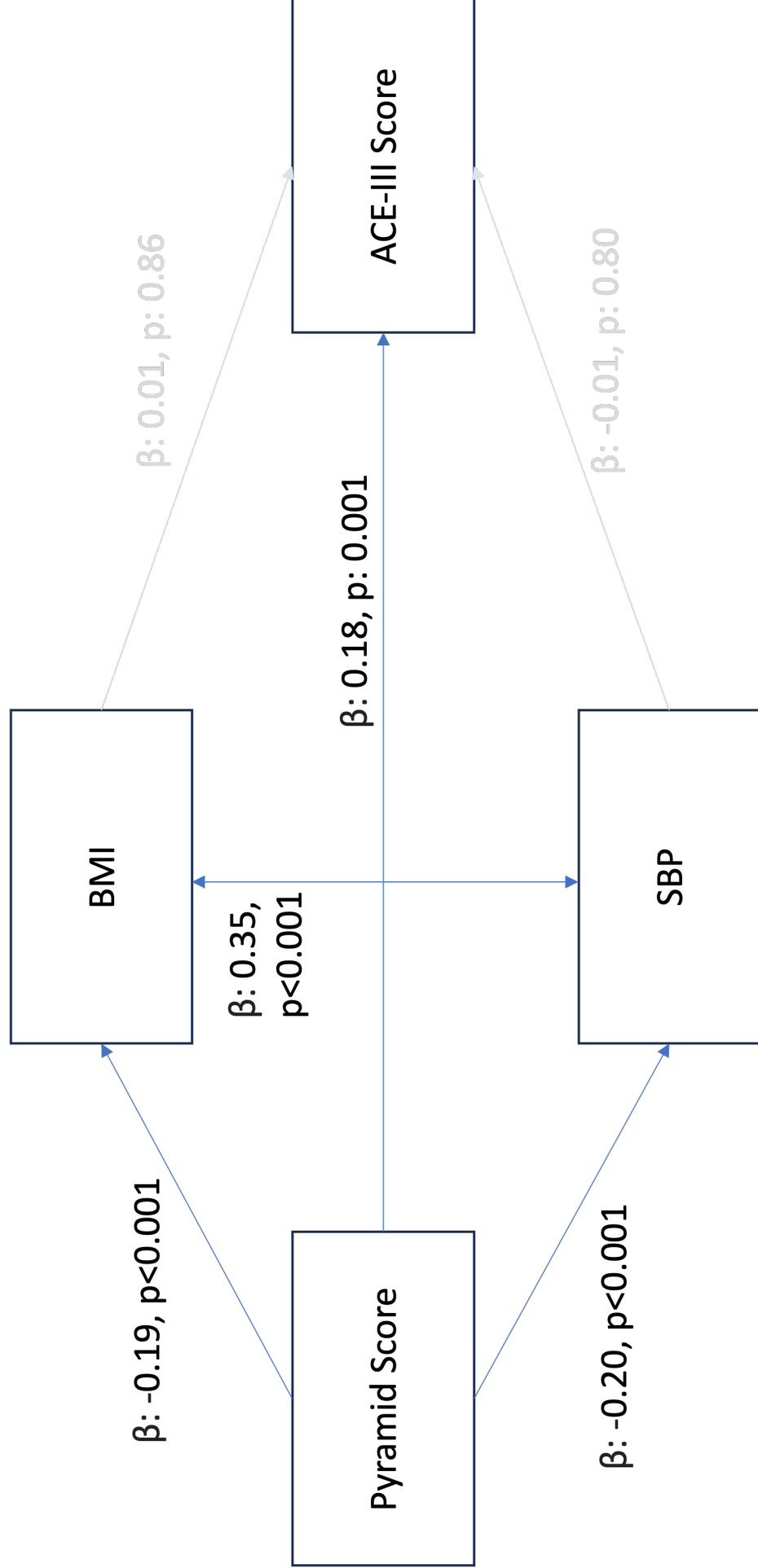

Supplement: Supplementary file 1 — APPENDIX S1: Supporting Information. [file ENE-31-e16345-s002.pdf]
