# Supplementary material for: The Mediterranean diet is not associated with neuroimaging or cognition in middle‐aged adults: a cross‐sectional analysis of the PREVENT dementia programme
Source: Eur J Neurol. 2024 May 25;31(8):e16345. doi: 10.1111/ene.16345 (PMC11236004; doi:10.1111/ene.16345)
Supplement: Supplementary file 2 — APPENDIX S2: Supporting Information. [file ENE-31-e16345-s001.docx]

| **Food component** | **MEDAS Rule** | | **MEDAS** | | **MEDAS-C Rule** | **MEDAS Continuous^±^** | | **Pyramid Rule** | | **Pyramid** | |
| --- | --- | --- | --- | --- | --- | --- | --- | --- | --- | --- | --- |
| Olive oil (consumption) | Rule 1 | | 1 point: Consumption  0 points: Non-consumption | | Rule 1 | 1 point: Consumption  0 points: Non-consumption | | Rule 15 | | 1 point: Consumption  0 points: Non-consumption | |
| Olive oil (volume)^¶^ | Rule 2 | | NA | | | | | | | | |
| Vegetables | Rule 3 | | 1 point: ≥2 portions/d  0 points: <2 portions/d | | Rule 3 | 1 point: ≥2 portions/d  0 points: 0 portions/d | | Rule 1 | | 1 point: ≥6 portions/d  0 points: 0 portions/d | |
| Fruit | Rule 4 | | 1 point: ≥3 portions/d  0 points: <3 portions/d | | Rule 4 | 1 point: ≥3 portions/d  0 points: 0 portions/d | | Rule 3 | | 1 point: 3-6portions/d  0 points: 0 portions/d  0.5 points: overconsumption^#^ | |
| Red meat | Rule 5 | | 1 point: <1 portion/d  0 points: ≥1 portion/d | | Rule 5 | 1 point: <1 portion/d  0 points: ≥2 portions/d | | Rule 8 | | 1 point: <2 portion/wk  0 points: ≥2 portions/wk | |
| Dairy | Rule 6 | | 1 point: <1 portion/d  0 points: ≥1 portion/d | | Rule 6 | 1 point: <1 portion/d  0 points: ≥2 portions/d | | Rule 6 | | 1 point: 1.2-2.5 portions/d  0 point: 0 portions  0.5: overconsumption^#^ | |
| Carbonated/sweet drinks | Rule 7 | | 1 point: <1 portion/d  0 points: ≥1 portion/d | | Rule 7 | 1 point: <1 portion/d  0 points: ≥2 portions/d | | NA | | | |
| Alcohol (wine only for MEDAS, MEDAS continuous) | Rule 8 | | 1 point: ≥7 portions/wk  0 points: <7 portions /wk | | Rule 8 | 1 point: ≥7 portions/wk  0 points: 0 portions /wk | | Rule 14 | | 1 point: 1.5-2.5 portions/d (male)  1 point: 0.5-1.5 portions/d (female)  0 points: 0 portions/d  0.5: overconsumption^#^ | |
| Pulses | Rule 9 | | 1 point: ≥3 portions/wk  0 points: <3 portions/wk | | Rule 9 | 1 point: ≥3 portions/wk  0 points: 0 portions/wk | | Rule 2 | | 1 point: ≥2 portions/wk  0 points: 0 portions/wk | |
| Fish/seafood | Rule 10 | | 1 point: ≥3 portions/wk  0 points: <3 portions/wk | | Rule 10 | 1 point: ≥3 portions/wk  0 points: 0 portions/wk | | Rule 7 | | 1 point: ≥2 portions/wk  0 points: 0 portions/wk | |
| Pastries | Rule 11 | | 1 point: <2 portions/wk  0 points: ≥2 portions/wk | | Rule 11 | 1 point: <2 portion/wk  0 points: ≥4 portions/wk | | NA | | | |
| Nuts | Rule 12 | | 1 point: ≥3 portions/wk  0 points: <3 portions/wk | | Rule 12 | 1 point: ≥3 portions/wk  0 points: 0 portions/wk | | Rule 4 | | 1 point: 1-2 portions/d  0 points: 0 portions/d  0.5 points: overconsumption^#^ | |
| White meat | Rule 13 | | 1 point: more white meat  0 points: more red meat | | Rule 13 | 1 point: more white meat  0 points: more red meat | | Rule 10 | | 1 point: 1.5-2.5 portions/wk  0 point: 0 portions/wk  0.5: overconsumption^#^ | |
| Sofrito | Rule 14 | | 1 point: ≥2 portions/wk  0 points: <2 portions/wk | | Rule 14 | 1 point: ≥2 portions/wk  0 points: 0 portions/wk | | NA | | | |
| Cereals | NA | | | | | | | Rule 5 | | 1 point: 3-6 portions/d  0 points: 0 portions/d  0.5 points: overconsumption^#^ | |
| Processed meat | NA | | | | | | | Rule 9 | | 1 point: <2 portion/wk  0 points: ≥2 portions/wk | |
| Eggs | NA | | | | | | | Rule 11 | | 1 point: 2-4 potions/wk  0 points: 0 portions/wk  0.5: overconsumption^#^ | |
| Potato | NA | | | | | | | Rule 12 | | 1 point: ≤3 portions/wk  0 points: >3 portions/wk | |
| Sweets | NA | | | | | | | Rule 13 | | 1 point: ≤2 portions/wk  0 points: >2 portions/wk | |
| **Supplementary Table S1:** Scoring criteria for MedDiet scores (MEDAS, MEDAS Continuous and Pyramid scores. ^¶^Data on volume of oil consumed not available, component excluded from all scores. ^±^Scores assigned on continuous scale between 0 and 1 with servings required for 0 and 1 indicated in table. ^#^Two-fold higher than mid-point of recommended intake. Abbreviations: Tbsp: Tablespoon; d: day; wk: week | | | | | | | | | | | |
| **Food Groups** | | | | | | | | | | |  |
| Cold breakfast cereals | | *Processed meats* | | Mayonnaise | | | *Potatoes* | | *Condiments* | |  |
| Low-fat dairy products | | Fish | | Oil and vinegar dressings | | | Cruciferous vegetables | | Nuts | |  |
| *Eggs* | | *French Fries* | | Legumes | | | Fruit | | Tea | |  |
| *Red meats* | | Whole grains | | Dark-yellow vegetables | | | *High-fat dairy products* | | Coffee | |  |
| Poultry | | Pizza | | Green leafy vegetables | | | *Snacks* | | Tomatoes | |  |
| *Organ meats* | | Other soups | | Other vegetables | | | *Refined grains* | | Low-energy drinks | |  |
| *High-energy drinks* | | *Sweets and desserts* | | Beer | | | Wine | | Liquor | |  |
| Butter and margarine | |  | |  | | |  | |  | |  |
| **Supplementary Table S2:** Table of food groups used for principal component analysis to create Western Diet Score. Food groups underlined and italicised loaded to the factor termed as the Western Diet. | | | | | | | | | | |  |

Figure Legend:

**Supplementary Figure S1:** Path analysis of associations between the Pyramid score, body mass index (BMI), systolic blood pressure (SBP) and Addenbrookes Cognitive Examination-III (ACE-III) total score.
